# Improved Neural Networks with Random Weights for Short-Term Load Forecasting

**DOI:** 10.1371/journal.pone.0143175

**Published:** 2015-12-02

**Authors:** Kun Lang, Mingyuan Zhang, Yongbo Yuan

**Affiliations:** Faculty of Infrastructure Engineering, Dalian University of Technology, Dalian, 116024, China; Universitat de Valencia, SPAIN

## Abstract

An effective forecasting model for short-term load plays a significant role in promoting the management efficiency of an electric power system. This paper proposes a new forecasting model based on the improved neural networks with random weights (INNRW). The key is to introduce a weighting technique to the inputs of the model and use a novel neural network to forecast the daily maximum load. Eight factors are selected as the inputs. A mutual information weighting algorithm is then used to allocate different weights to the inputs. The neural networks with random weights and kernels (KNNRW) is applied to approximate the nonlinear function between the selected inputs and the daily maximum load due to the fast learning speed and good generalization performance. In the application of the daily load in Dalian, the result of the proposed INNRW is compared with several previously developed forecasting models. The simulation experiment shows that the proposed model performs the best overall in short-term load forecasting.

## Introduction

As with water supply, gas supply, communications, and transportation systems, electric power system is a necessary component of the urban lifeline engineering as well. Accurate load forecasting is increasingly important since it is critical for the planning, operations and investments of power systems [1]. Improving the accuracy of load forecasting contributes to the promotion of the power supply efficiency and the reduction of operating costs [2].

Load forecasting can be classified into long-term, mid-term, short-term and very short-term forecasting, based on the forecasting horizon. During the past decades, researchers have developed many different kinds of methods to improve the load forecasting accuracy [1], especially in the field of short-term load forecasting [3–5]. Most of these methods have been restricted in practical applications due to the randomness and nonlinearity of the short-term load. In contrast, some intelligent forecasting calgorithms, such as artificial neural network (ANN) [6,7] and support vector machine (SVM), have been widely used [2]. Park et al. first used ANN to forecast short-term load [8]. Lee et al. analyzed the influence of different structures of the ANN on forecasting results [5]. Hippert et al gave a review of ANN methods for short-term load forecasting, and pointed out the overfitting problems existing in ANN methods [4]. Taylor et al. took the weather into account while modeling with ANN methods [9]. In addition, SVM performs well in the field of short-term load forecasting as well. Moreover, as SVM is based on the structural risk minimization framework, it can overcome overfitting problems effectively [10]. However, the effectiveness of SVM depends on the selection of kernel, the kernel's parameters, and the regularization parameter. Typically, each combination of parameters is checked using the cross validation, and the best combination of parameters is often selected by the grid search method with the exponentially growing computational complexity. Simulated annealing algorithm [11], genetic algorithm [12] and particle swarm optimization were used by some researchers to select the proper parameters of SVM.

Recently, researchers from all over the world have been improving the ANN according to different forecasting tasks and have obtained some satisfying results [13]. Nevertheless, the gradient-based learning algorithms are widely used to train traditional ANNs, which may result in some drawbacks such as the slow convergence speed, the local minimum, and the overfitting phenomenon. In order to solve the aforementioned problems, we focus our study in this paper on an improved machine learning algorithms based on neural networks with random weights (NNRW) models [14]. There are three layers in NNRW: input layer, hidden layer, and output layer. In the NNRW, the weights connecting the input layer to the hidden layer, as well as the bias values of the hidden layer, are randomly generated before the learning process. Only the weights connecting the hidden layer to the output layer are trained by the fast linear regression. Because of the rapid learning speed and the good generalization performance, NNRW has been successfully used in fields of computational intelligence and machine learning communities, such as electricity price forecasting [15], power loss analysis [16], lying and truth-telling classification [17], and attention-deficit/hyperactivity disorder (ADHD) classification [18]. The structure of NNRW, i.e. the number of the hidden nodes, is one of the important factors that affect the performance of NNRW. It is empirically determined by the users. Recently, neural networks with random weights and kernels (KNNRW) [19–22] has been proposed by replacing the hidden nodes mapping with the kernel mapping. It does not need to determine the number of hidden nodes of KNNRW.

Based on the analysis above, this paper proposes a short-term load forecasting method based on KNNRW, which can combine the fast learning speed of NNRW and the good generalization performance of SVM. Eight relevant factors (e.g., the historical load data, the temperature data, and the holiday data) are first selected as the inputs of the forecasting model. It is known that the inputs are treated equally in KNNRW. However, different inputs may have different influences on the forecasting values. As a result, a mutual information weighting algorithm is then applied to allocate different weights to the inputs according to the corresponding influences. Finally, the resulting improved neural networks with random weights is used to approximate the nonlinear function between the selected inputs and the daily maximum load.

## Neural Networks with Random Weights and Kernels

### 2.1 Basic Neural Networks with Random Weights

NNRW has been proposed by Schmidt et al. [14]. However, there are still existing some similar ideas coming out from other researchers, such as Pao et al. [23] and Huang et al. [24]. Pao et al. described such randomized learner models as the random vector functional-link (RVFL) net [23]. Huang et al. defined such machine learning models as extreme learning machine (ELM) [24]. Researchers have done some further researches on RVFL and ELM, and achieved some theoretical results [22,25,26]. In fact, a feed forward NNRW has a simple three-layer structure: input layer, output layer, and a hidden layer consisting of a large number of nonlinear processing nodes. Mathematically, NNRW [14] can be expressed as follows:

$$o_{k}=w^{\text{T}}g\;\left(W_{in}\cdot x_{k}+b\;\right),k=1,2,\ldots ,N$$ (1)

where ***W***_*in*_ ∈ *R* ^*L*×*m*^ is the input weight matrix, ***b*** ∈ *R* ^*L*^ is the bias value vector of the hidden layer, ***w*** ∈ *R* ^*L*^ is the output weight vector, *g*(⋅) is the activation function (*g*(⋅) could be almost any nonlinear piecewise continuous activation function or any linear combination of these functions), *N* is the number of the samples, *L* is the number of the hidden layer nodes, ***x***_*k*_ ∈ *R* ^*m*^ is the input vector which has m-dimension features, and *o*_*k*_ ∈ *R* is the output value.

The output of the proposed forecasting model is the maximum load of the next day. Consequently, we use the single output form of NNRW in this paper.

For *N* arbitrary distinct samples {***x***_*i*_ ∈ *R* ^*n*^, *t*_*i*_ ∈ *R*}, NNRW with *L* hidden nodes can approximate these *N* samples with zero error. It means that $\sum_{k=1}^{N}\left\|o_{k}-t_{k}\right\|=0$, i.e., there exists ***w*** in NNRW such that

$$w^{\text{T}}g\left(W_{in}\cdot x_{k}+b\right)=t_{k},k=1,2,\ldots ,N$$ (2)

The matrix-vector formulation of (2) can be written as

Hw=t (3)

where $H={\left[\begin{array}{ccc} g\left(W_{in}\cdot x_{1}+b_{1}\right) & \cdots & g\left(W_{in}\cdot x_{1}+b_{n}\right)\\ \vdots & \ddots & \vdots \\ g\left(W_{in}\cdot x_{N}+b_{1}\right) & \cdots & g\left(W_{in}\cdot x_{N}+b_{n}\right) \end{array}\right]}_{N\times L}$ is the hidden layer output matrix of NNRW, and ***t*** = [*t*_1_, *t*_2_,…,*t*_*N*_]^*T*^ is the desired output vector.

In the NNRW model, ***W***_*in*_ and ***b*** are generated randomly beforehand, and remain fixed in the training process. ***w*** is the only parameter that needs to be tuned through the training. It can be calculated analytically as follows:

$$w=H^{†}T$$ (4)

where ***H*** ^†^ is the Moore-Penrose generalized inverse of matrix ***H***.

The training of the NNRW model can be summarized as follows:

- Randomly generate the input weight ***W***_*in*_ and the hidden layer bias ***b***;
- Calculate the hidden layer output matrix ***H***;
- Calculate the output weight ***w*** by (4).

As can be seen from the above, the training process of NNRW is a simple linear regression process, which can overcome the limitations of traditional ANNs effectively. Despite the success of NNRW, there is still room for improvement, such as the determination of the structure (i.e., the number of the hidden layer nodes), and the ill-conditioned solution in the training process [22].

### 2.2 Neural Networks with Random Weights and Kernels

In order to overcome the aforementioned shortcomings of NNRW, neural networks with weights and kernels (KNNRW) has been proposed by introducing the kernel function mapping of SVM as the hidden node mapping of NNRW [19,21].

The optimization problem of NNRW can be written as:

$$\begin{array}{l} \text{min}\;L_{P_{NNRW}}=\frac{1}{2}{\left\|w\right\|}^{2}+\frac{C}{2}\sum_{i=1}^{N}{\left\|\xi_{i}\right\|}^{2}\\ \text{s}.\text{t}.\;h\left(x_{i}\right)\cdot w=t_{i}-\xi_{i},i=1,\ldots ,N \end{array}$$ (5)

where *ξ*_*i*_ is the training error related to the *i*th training sample ***x***_*i*_, *C* is the regularization coefficient, and ***h***(***x***_*i*_) denotes the *i*th row of ***H***. The corresponding dual optimization problem of (5) can be formulated as:

$$L_{D_{NNRW}}=\frac{1}{2}{\left\|w\right\|}^{2}+\frac{C}{2}\sum_{i=1}^{N}{\left\|\xi_{i}\right\|}^{2}-\sum_{i=1}^{N}\alpha_{i}\left(\;h\left(x_{i}\right)\cdot w-t_{i}+\xi_{i}\right)$$ (6)

where *α*_*i*_ is the Langrage multiplier with respect to the *i*th training sample ***x***_*i*_. The corresponding Karush-Kuhn-Tucker (KKT) conditions are as follows:

$$\frac{\partial L_{D_{NNRW}}}{\partial w}=0\to w=\sum_{i=1}^{N}\alpha_{i}h{\left(x\right)}^{T}\to w=H^{T}\alpha$$ (7)

$$\frac{\partial L_{D_{NNRW}}}{\partial \xi_{i}}=0\to \alpha_{i}=C\xi_{i},i=1,\ldots ,N$$ (8)

$$\frac{\partial L_{D_{NNRW}}}{\partial \alpha_{i}}=0\to h\left(x_{i}\right)\cdot w-t_{i}+\xi_{i}=0,i=1,\ldots ,N$$ (9)

Substituting (7) and (8) into (9), the following equation can be obtained

$$\left(\frac{I}{C}+{\mathrm{HH}}^{T}\right)\alpha =T$$ (10)

where ***I*** is an identity matrix.

Considering (7) and (10), the weight ***w*** can be calculated as:

$$w=H^{T}{\left(\frac{I}{C}+{\mathrm{HH}}^{T}\right)}^{-1}T$$ (11)

Thus, the output function of NNRW can be written as:

$$f\left(x\right)=h\left(x\right)H^{T}{\left(\frac{I}{C}+{\mathrm{HH}}^{T}\right)}^{-1}T$$ (12)

It can be seen from (12) that the specific form of ***h***(***x***) is not important as long as the dot product of ***HH*** ^*T*^ (or ***h***(***x***)***H*** ^*T*^) is known. As a result, if the hidden node mapping ***h***(***x***) is unknown, we can define the kernel matrix of KNNRW as follows:

$$\begin{array}{l} \Omega_{NNRW}={\mathrm{HH}}^{T}\;:\\ \Omega_{NNRWi,j}=h\left(x_{i}\right)\cdot h\left(x_{j}\right)=K\left(x_{i},x_{j}\right) \end{array}$$ (13)

Consequently, the output function can be rewritten accordingly as:

$$f\left(x\right)={\left[\begin{array}{c} K\left(x,x_{1}\right)\\ \vdots \\ K\left(x,x_{N}\right) \end{array}\right]}^{T}{\left(\frac{I}{C}+\Omega_{NNRW}\right)}^{-1}T$$ (14)

In the kernel implementation of NNRW, ***h***(***x***) can be unknown, while the corresponding kernel function *K* (***u***, ***v***) usually should be given (e.g., *K* (***u***, ***v***) = exp(−*γ*‖***u***−***v***‖^2^), where *γ* is the kernel width.). Hence, the number of the hidden layer nodes does not need to be determined any more. Moreover, the KNNRW has the following universal approximation capability:

*Theorem* [21]: Universal Approximation Capability: According to NNRW, a widespread type of the hidden node mapping ***h***(***x***) can be used in NNRW so that NNRW can approximate any continuous target function. In other words, given any target continuous function *g*(***x***), there is a weight vector ***w*** such that

$$\underset{L\to +\infty }{\text{lim}}\left\|f\left(x\right)-g\left(x\right)\right\|=\underset{L\to +\infty }{\text{lim}}\left\|\sum_{i=1}^{L}w_{i}h\left(x\right)-g\left(x\right)\right\|=0$$ (15)

With this universal approximation capability, KNNRW can use a wide range of feature mappings, such as Sigmoid, radial basis function (RBF), trigonometric, and polynomial mappings. The optimization objective functions of KNNRW are similar to those of traditional SVM/least squares support vector machine (LS-SVM). However, KNNRW does not have any constraints on the Lagrangian multipliers. As a result, KNNRW can obtain a better solution than SVM/LS-SVM. In addition, as KNNRW does not need the bias values while SVM does need, it is superior to the traditional SVM/LS-SVM algorithms in the performance of the scalability and learning rate [21].

## Short-Term Load Forecasting Model Based on KNNRW

### 3.1 Inputs of KNNRW

**Fig 1:**
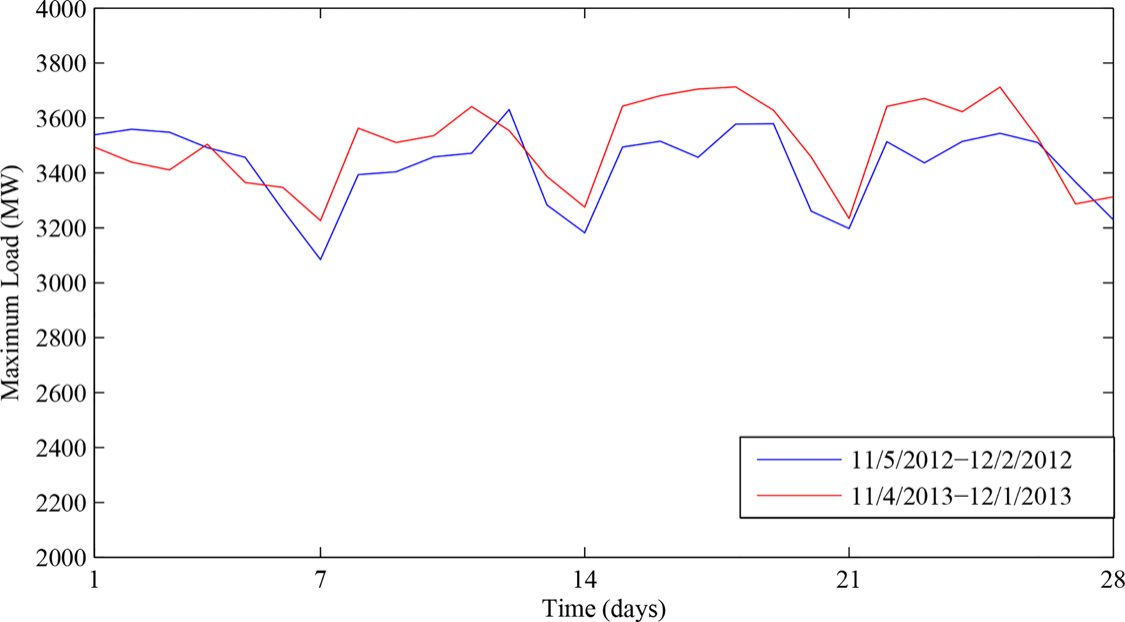
The week characteristic of daily maximum load. The blue line represents the maximum load from November 5th of 2012 to December 2nd of 2012. The red line represents the maximum load from November 4th of 2013 to December 1st of 2013.

**Fig 2:**
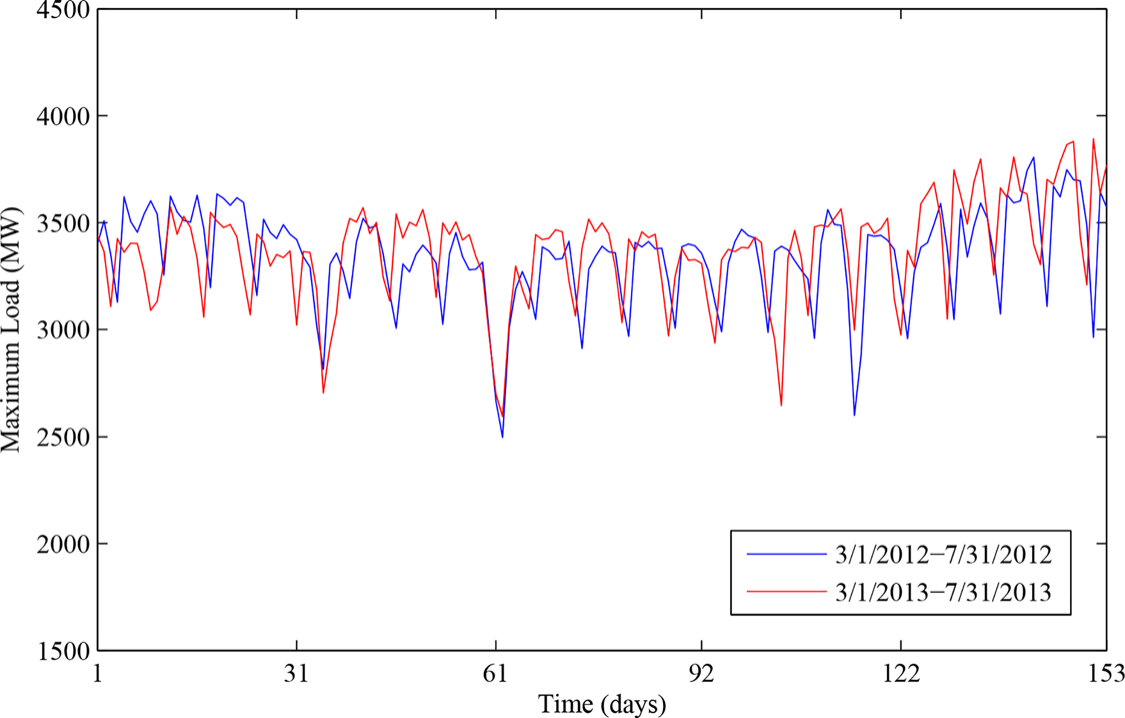
The month characteristic of daily maximum load. The blue line represents the maximum load from March 1st of 2012 to July 31st of 2012. The red line represents the maximum load from March 1st of 2013 to July 31st of 2013.

In this section, the proposed INNRW was used to forecast the short-term load of Dalian city of China. The output of the model was the daily maximum load. With the analysis in literature [4], the load data had weekly and monthly characteristics. It can be seen from Figs 1 and 2 that values of the load remain stable on weekdays while dropping apparently at weekends; values of the same month show approximately the same tendency; values of every week indicate a regular variation tendency (Take Dalian as an example). Therefore, we took both weekly and monthly characteristics as the inputs. Additionally, it was verified that the temperature was an essential factor influencing the maximum load [9], and the temperature showed an obvious correlation with the maximum load. Therefore, we selected the temperature as another input.

**Fig 3:**
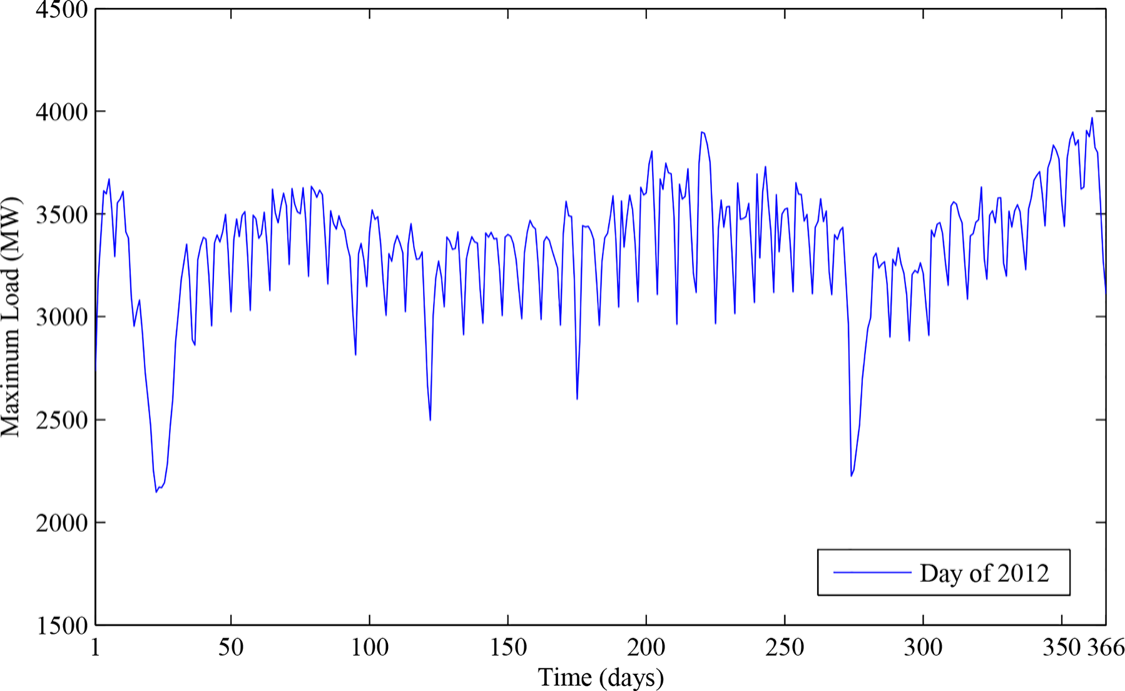
The holiday characteristic of daily maximum load. The blue line represents the maximum load of 2012.

Meanwhile, the holiday data also affected the maximum load, for the descent of the industries power consumptions during the holidays can lead to the decrease of the total power consumptions. For example, as is known, there were 6 Chinese legal holiday vacations in 2012, and they were from 1st January to 3rd January, from 22nd January to 28th January, from 2nd April to 4th April, from 29th April to 1st May, from 22nd June to 24th June, and from 30th September to 7th October, respectively. In addition, it can be clearly seen from Fig 3 that the load data have an obvious holiday characteristic, that is, values of the load descend sharply during the holidays. Consequently, the binary encoded holiday data served as an input in this paper. As the maximum load was closely related to the historical maximum load, which can be verified by analyzing the load data as time series, we selected the maximum load of the day before, and that of the day last week as inputs of KNNRW.

Finally, the inputs selected for the INNRW were month of the year, day of the month, day of the week, week number, holiday indicator, daily average temperature, maximum electricity load of the day before, and maximum electricity load of the day last week.

### 3.2 Mutual Information Weighting Algorithm

In order to further improve the forecasting accuracy, the contributions of the inputs to the output of KNNRW were calculated and the weight values were allocated to the inputs accordingly. The mutual information (MI) is a measurement of the variables’ mutual dependence [27–30]. Accordingly, the high mutual information indicates the high dependence, and the low mutual information indicates the low dependence.

For two given discrete variables *X* and *Y*, suppose the joint probability distribution was *P*_*XY*_(*x*, *y*), and the mutual information between *X* and *Y*, denoted *I*(*X*;*Y*), can be formatted as

$$I(X;Y)=\sum_{x,y}P_{XY}(x,y)\text{log}\frac{P_{XY}(x,y)}{P_{X}(x)P_{Y}(Y)}$$ (16)

where *P*_*X*_(*x*) and *P*_*Y*_(*y*) were the marginal probability distribution

$$\begin{array}{l} P_{X}(x)=\sum_{y}P_{XY}(x,y)\\ P_{Y}(y)=\sum_{x}P_{XY}(x,y) \end{array}$$ (17)

In the case of continuous variables, (16) was replaced by

$$I(X;Y)=\int_{Y}\int_{X}P_{XY}(x,y)\text{log}\frac{P_{XY}(x,y)}{P_{X}(x)P_{Y}(Y)}dxdy$$ (18)

where *P*_*XY*_(*x*, *y*) was the joint probability density function of *X* and *Y*, and *P*_*X*_(*x*) and *P*_*Y*_(*y*) were the marginal probability density functions of *X* and *Y*, respectively.

For discrete feature variables, both the joint and marginal probability can be estimated by tallying the samples of the categorical variables in the data. For continuous feature varibles, the following Parzen windows method was used to approxiamte *I*(*X*;*Y*).

Given *N* samples of a vector variable ***x***, the approximate density funciton ${\hat{P}}_{X}(x)$ had the following form:

$${\hat{P}}_{X}(x)=\frac{1}{N}\sum_{i=1}^{N}\delta \left(x-x^{\left(i\right)},h\right)$$ (19)

where ***x*** ^(*i*)^ was the *i*th sample, *h* was the window width, and *δ*(⋅) was the Parzen window function:

$$\delta \left(z,h\right)=\text{exp}\left(-\frac{z^{T}\Sigma^{-1}z}{2h^{2}}\right)/\left\{{\left(2\pi \right)}^{d/2}h^{d}{\left|\Sigma \right|}^{1/2}\right\}$$ (20)

where ***z*** = ***x*** − ***x*** ^(*i*)^, *d* was the dimension of the sample ***x*** and Σ was the covariance of ***z***. When *d* = 1, (19) returned the estimated marginal density; when *d* = 2, we can use (19) to estimate the density of the bivariate (*x*, *y*), *P*_*XY*_ (*x*, *y*), which was the joint density of *x* and *y* in fact.

Hence, in this paper, we used the mutual information to determine the contribution of the inputs to the output of the INNRW. First, the mutual information *MI*_*i*_, *i* = 1,…, *m* of the inputs to the output were calculated. Then the weights can be allocated to the corresponding inputs according to the following equation

$$\mu_{i}=\frac{{MI}_{i}}{\sum_{i}{MI}_{i}}$$ (21)

where *μ*_*i*_ was the weight allocated to the *i*th input. Then, the input of KNNRW can be expressed as $x_{j}={\left[\;\begin{array}{cccc} \mu_{1}x_{1j} & \mu_{2}x_{2j} & \cdots & \mu_{m}x_{mj} \end{array}\;\right]}^{T}$. And the resulting forecasting model was denoted as the improved neural networks with random weights.

## Simulation

In order to verify the effectiveness, the proposed model was applied to forecast the actual maximum load. The electricity load data from January 1, 2012 to November 30, 2013 from the Dalian Electricity Corporation in China, the temperature, the holiday indicator and some other data were used to train the forecasting model. Daily maximum load data of 31 days in December of 2013 were used to test the performance of the forecasting model. The forecasting results were described using Mean Absolute Percentage Error (MAPE), Maximum Error (ME) and Forecasting Error (FE) as follows:

$$\text{MAPE}=100\cdot \frac{\sum_{i=1}^{n}\left|\frac{L_{R_{i}}-L_{P_{i}}}{L_{R_{i}}}\right|}{n}$$ (22)

$$\text{ME}=\text{max}\left(\left|L_{R_{i}}-L_{P_{i}}\right|\right),i=1,\ldots ,n$$ (23)

$$\text{FE}=\frac{L_{P_{i}}-L_{R_{i}}}{L_{R_{i}}}\cdot 100\%,i=1,\ldots ,n$$ (24)

where $L_{R_{i}}$ stood for the actual values of the daily maximum load, $L_{P_{i}}$ stood for the forecasting values of the daily maximum load, and *n* stood for the number of days.

### 4.1 Simulation Experiment

Firstly, data sets were normalized. The inputs were normalized to [–1, 1] and the outputs were normalized to [0, 1]. According to (18), the weights were calculated and allocated to the corresponding inputs.

Secondly, the INNRW model was initialized, in which the Gaussian kernel function was used in the hidden layer, and the regularization coefficient and kernel width were determined by the grid search.

Thirdly, the INNRW model was trained by the training samples.

Fourthly, the testing samples based on the trained INNRW were forecasted, and the forecasting results of the daily maximum load of 31 days in December of 2013 were obtained.

Eventually, the residual errors between predicted values and actual values were calculated.

### 4.2 Experiment Results

Based on Eq (18), the mutual information and the resulting weights of the inputs are summarized in Table 1.

**Table 1:** Mutual information and weight results.

Input Feasures	Month of the Year	Day of the Month	Day of the Week	Week Number	Holiday Indica-tor	Daily Average Temper-ature	Maximum Electricity Load of the Day Before	Maximum Electricity Load of the Day Last Week
MI	0.0286	0.0060	0.0402	0.0075	0.1291	0.0652	0.2854	0.0932
Weight	0.0437	0.0092	0.0614	0.0114	0.1970	0.0995	0.4356	0.1422

**Fig 4:**
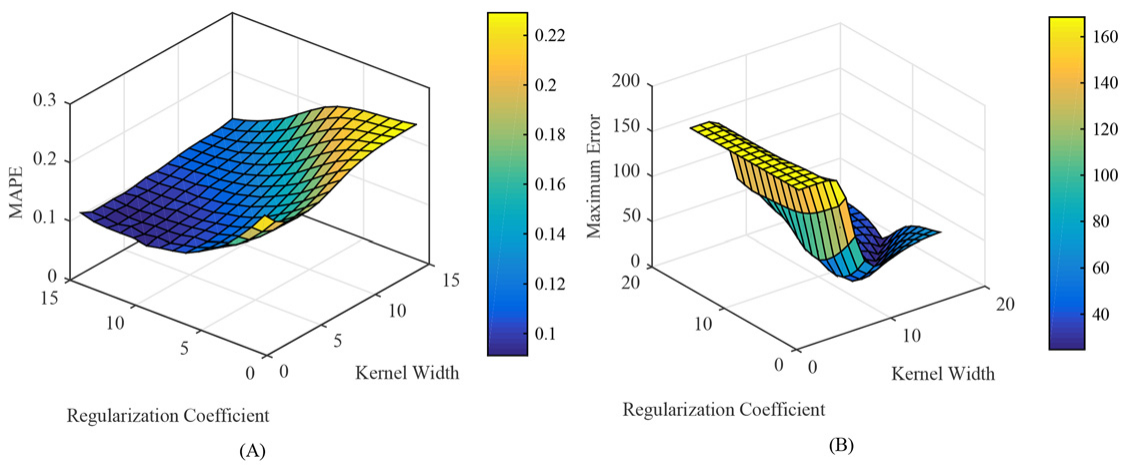
Kernel width and regularization coefficient cross validation. (A) Kernel width and regularization coefficient of MAPE. (B) Kernel width and regularization coefficient of ME.

Based on the analysis above, the Gaussian kernel function *K*(***u***, ***v***) = exp(−*γ*‖***u***−***v***‖^2^), where *γ* was the kernel width, was chosen to be the kernel function in the INNRW model. Fig 4(A) illustrates the relations among MAPE, the kernel width and the regularization coefficient, while Fig 4(B) illustrates the relations among ME, the kernel width and the regularization coefficient. It can be seen from Fig 4 that both the kernel width and the regularization parameter are key parameters influencing the forecasting performance of the INNRW. The grid search method was used to optimize the two parameters. The optimal kernel width was 3.7276e+03, and the optimal regularization parameter was 1.3895.

In order to further illustrate the effectiveness of the proposed method, a comparison was conducted between the INNRW method and several state-of-the-art load forecasting methods, such as back propagation (BP) neural network, RBF neural network, support vector regression (SVR), NNRW, online sequential extreme learning machine (OS-ELM) and KNNRW. The forecasting results were shown in Table 2, Table 3 and Table 4.

**Table 2:** Forecasting results.

Day of December	Actual Load (MW)	BP			SVR		
		Forecasting Load(MW)	Forecasting ME(MW)	Forecasting Error (%)	Forecasting Load(MW)	Forecasting ME(MW)	Forecasting Error (%)
1	3312.8	3077.9	234.9	-7.09	3270.1	42.7	-1.29
2	3520.2	3548.9	28.7	0.82	3452.8	67.4	-1.92
3	3615.5	3577.9	37.6	-1.04	3481.0	134.5	-3.72
4	3541.8	3588.7	46.9	1.32	3545.8	4.0	0.11
5	3600.4	3535.5	64.9	-1.80	3585.7	14.7	-0.41
6	3533.1	3522.2	10.9	-0.31	3546.9	13.8	0.39
7	3444.5	3380.3	64.2	-1.86	3431.8	12.7	-0.37
8	3304.3	3217.6	86.7	-2.62	3254.5	49.8	-1.51
9	3610.8	3577.7	33.1	-0.92	3625.9	15.1	0.42
10	3683.2	3656.4	26.8	-0.73	3673.8	9.4	-0.26
11	3744.3	3708.0	36.3	-0.97	3774.3	30.0	0.80
12	3725.7	3717.4	8.3	-0.22	3809.0	83.3	2.24
13	3779.4	3697.5	81.9	-2.17	3760.1	19.3	-0.51
14	3633.8	3646.8	13	0.36	3582.4	51.4	-1.41
15	3554.6	3429.5	125.1	-3.52	3362.3	192.3	-5.41
16	3799.0	3666.2	132.8	-3.50	3792.5	6.5	-0.17
17	3768.9	3730.7	38.2	-1.01	3842.7	73.8	1.96
18	3757.3	3779.3	22	0.59	3969.8	212.5	5.65
19	3828.4	3785.7	42.7	-1.12	3954.8	126.4	3.30
20	3839.8	3794.2	45.6	-1.19	3862.5	22.7	0.59
21	3638.9	3751.2	112.3	3.09	3652.4	13.5	0.37
22	3570.3	3497.4	72.9	-2.04	3389.4	180.9	-5.07
23	3833.7	3656.5	177.2	-4.62	3699.5	134.2	-3.50
24	3884.6	3738.0	146.6	-3.77	3797.5	87.1	-2.24
25	3840.4	3726.4	114	-2.97	3820.6	19.8	-0.52
26	3867.6	3795.7	71.9	-1.86	3906.7	39.1	1.01
27	3904.8	3803.1	101.7	-2.60	3795.9	108.9	-2.79
28	3705.9	3777.1	71.2	1.92	3600.3	105.6	-2.85
29	3495.5	3495.6	0.1	0.00	3285.6	209.9	-6.00
30	3561.9	3605.0	43.1	1.21	3340.6	221.3	-6.21
31	3333.9	3575.2	241.3	7.24	3311.8	22.1	-0.66

**Table 3:** Forecasting results.

Day of December	Actual Load (MW)	OS-ELM			INNRW		
		Forecasting Load(MW)	Forecasting ME(MW)	Forecasting Error (%)	Forecasting Load(MW)	Forecasting ME(MW)	Forecasting Error (%)
1	3312.8	3277.2	35.6	-1.07	3146.0	166.8	-5.04
2	3520.2	3540.6	20.4	0.58	3504.5	15.7	-0.45
3	3615.5	3558.4	57.1	-1.58	3544.0	71.5	-1.98
4	3541.8	3612.5	70.7	2.00	3594.7	52.9	1.49
5	3600.4	3640.3	39.9	1.11	3528.8	71.6	-1.99
6	3533.1	3602.3	69.2	1.96	3516.9	16.2	-0.46
7	3444.5	3485.9	41.4	1.20	3394.0	50.5	-1.47
8	3304.3	3282.7	21.6	-0.65	3233.0	71.3	-2.16
9	3610.8	3609.1	1.7	-0.05	3509.9	100.9	-2.79
10	3683.2	3706.2	23.0	0.62	3649.8	33.4	-0.91
11	3744.3	3788.9	44.6	1.19	3705.6	38.7	-1.03
12	3725.7	3842.3	116.6	3.13	3719.0	6.7	-0.18
13	3779.4	3805.5	26.1	0.69	3677.1	102.3	-2.71
14	3633.8	3655.3	21.5	0.59	3628.1	5.7	-0.16
15	3554.6	3416.2	138.4	-3.89	3447.2	107.4	-3.02
16	3799.0	3844.4	45.4	1.20	3654.1	144.9	-3.82
17	3768.9	3868.0	99.1	2.63	3785.3	16.4	0.44
18	3757.3	3960.2	202.9	5.40	3794.9	37.6	1.00
19	3828.4	3942.2	113.8	2.97	3762.8	65.6	-1.71
20	3839.8	3880.3	40.5	1.06	3757.8	82.0	-2.13
21	3638.9	3682.7	43.8	1.20	3699.0	60.1	1.65
22	3570.3	3419.0	151.3	-4.24	3469.3	101.0	-2.83
23	3833.7	3702.7	131.0	-3.42	3636.5	197.2	-5.14
24	3884.6	3777.0	107.6	-2.77	3809.4	75.2	-1.94
25	3840.4	3757.0	83.4	-2.17	3802.8	37.6	-0.98
26	3867.6	3919.7	52.1	1.35	3790.1	77.5	-2.00
27	3904.8	3841.0	63.8	-1.63	3767.9	136.9	-3.51
28	3705.9	3675.9	30.0	-0.81	3722.6	16.7	0.45
29	3495.5	3335.4	160.1	-4.58	3452.2	43.3	-1.24
30	3561.9	3450.6	111.3	-3.12	3528.7	33.2	-0.93
31	3333.9	3377.1	43.2	1.30	3518.4	184.5	5.54

The forecasting results of BP neural network, SVR, OS-ELM and the proposed INNRW were summarized in Table 2 and Table 3. It can be observed from Table 2 and Table 3 that the ME of BP neural network is 241.3 and the maximum FE is 7.24%; the ME of SVR is 221.5 and the maximum FE is -6.21%; the ME of OS-ELM is 202.9 and the maximum FE is 5.40%; while the ME of the proposed INNRW is 184.5 and the maximum FE is 5.54%. The mean absolute forecasting error of the INNRW is 1.97%. There are 9 days’ forecasting errors of the INNRW are less than 1.00%, and 19 days’ are less than 2.00%. Moreover, the training time of the INNRW is much smaller than BP and SVR.

**Table 4:** Compared forecasting results.

Methods	MAPE	ME
BP	2.6787	241.2887
RBF	1.8728	316.8827
SVR	2.0537	221.2840
NNRW	1.7404	210.5358
OS-ELM	1.9408	202.8823
KNNRW	1.7195	209.2874
INNRW	**1.6944**	**184.5290**

**Fig 5:**
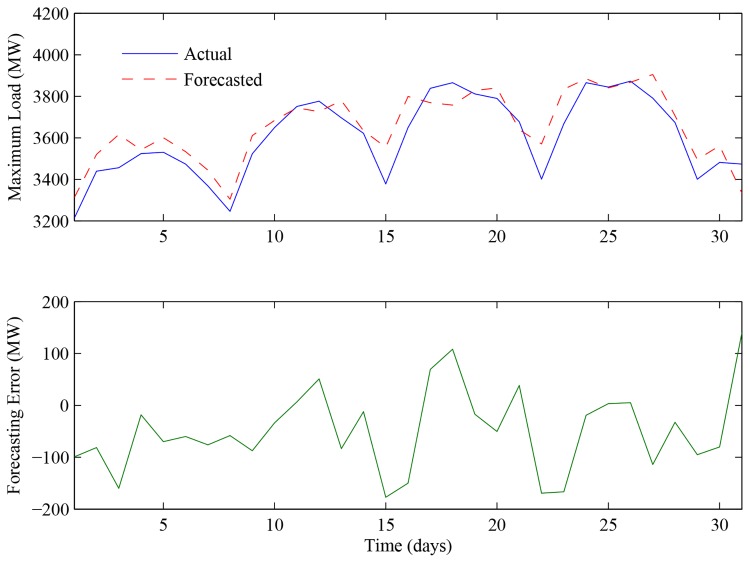
Forecasting results of daily maximum load of December in 2013. The blue solid line represents the forecasting maximum load of December in 2013. The red dotted line represents the actual maximum load of December in 2013. The green line represents the forecasting error between the forecasting values and the actual values.

As can be seen from Table 4, KNNRW and the proposed INNRW can obtain much better forecasting results in both MAPE and ME indexes than the other methods. Moreover, the INNRW outperforms KNNRW in both indexes, which demonstrates the effectiveness of the weighting algorithm. The forecasting results of December in 2013 based on the INNRW were described in Fig 5. It is clear that the predicted values can be approximately fitting the tendency of the actual values. Consequently, the effectiveness of the proposed method was well verified.

## Conclusions

A forecasting model based on the INNRW was proposed for the short-term load forecasting. Through the data pre-processing, eight features, i.e. month of the year, day of the month, day of the week, week number, holiday indicator, daily average temperature, maximum electricity load of the day before, and maximum electricity load of the day last week, were selected as the inputs of the INNRW. Then, in order to further improve the forecasting accuracy, different weights were allocated to the inputs according to their mutual information with the forecasting load values. A novel neural network, KNNRW, which combined the universal approximation ability and the fast learning speed of NNRW and the good generalization performance of SVM, was used to model the nonlinear function between the selected inputs and the maximum load. Simulation experiment results based on the actual load data from Dalian, China, showed that the proposed method can obtain smaller predicted errors than the traditional forecasting methods in both MAPE and ME.

The kernel types and kernel parameters were crucial to the forecasting performance of the INNRW, and they were selected by the time-consuming grid search in this paper. The multiple kernel learning will be a potential solution. It is able to combine the kernel funtions which have different types or different parameters. As a result, the investigation of the multiple kernel learning in the INNRW will be a subject of the further research.

## Supporting Information

S1 Matlab Codes (RAR)

## References

[1] Espinoza M, Suykens JA, Belmans R, De Moor B. Electric load forecasting. Control Systems, IEEE 2007, 27, 43–57.

[2] Ortiz-Arroyo D, Skov MK, Huynh Q. In *Accurate electricity load forecasting with artificial neural networks*, Computational Intelligence for Modelling, Control and Automation, 2005 and International Conference on Intelligent Agents, Web Technologies and Internet Commerce, International Conference on, 2005; IEEE: pp 94–99.

[3] Yan J, Li K, Bai E-W. In *Prediction error adjusted gaussian process for short-term wind power forecasting*, Intelligent Energy Systems (IWIES), 2013 IEEE International Workshop on, 2013; IEEE: pp 173–178.

[4] Hippert HS, Pedreira CE, Souza RC. Neural networks for short-term load forecasting: A review and evaluation. Power Systems, IEEE Transactions on 2001, 16, 44–55.

[5] Lee K, Cha Y, Park J. Short-term load forecasting using an artificial neural network. Power Systems, IEEE Transactions on 1992, 7, 124–132.

[6] Yu X, Efe MO, Kaynak O. In *A backpropagation learning framework for feedforward neural networks*, IEEE INTERNATIONAL SYMPOSIUM ON CIRCUITS AND SYSTEMS, 2001; Citeseer: pp 700–702.

[7] Yu X, Efe MO, Kaynak O. A general backpropagation algorithm for feedforward neural networks learning. Neural Networks, IEEE Transactions on 2002, 13, 251–254. 10.1109/72.977323 18244427

[8] Park DC, El-Sharkawi M, Marks R, Atlas L, Damborg M. Electric load forecasting using an artificial neural network. Power Systems, IEEE Transactions on 1991, 6, 442–449.

[9] Taylor JW, Buizza R. Neural network load forecasting with weather ensemble predictions. Power Systems, IEEE Transactions on 2002, 17, 626–632.

[10] Chen B-J, Chang M-W, Lin C-J. Load forecasting using support vector machines: A study on eunite competition 2001. Power Systems, IEEE Transactions on 2004, 19, 1821–1830.

[11] Pai P-F, Hong W-C. Support vector machines with simulated annealing algorithms in electricity load forecasting. Energy Conversion and Management 2005, 46, 2669–2688.

[12] Pai P-F, Hong W-C. Forecasting regional electricity load based on recurrent support vector machines with genetic algorithms. Electric Power Systems Research 2005, 74, 417–425.

[13] He Z, Hu Q, Zi Y, Zhang Z, Chen X. Hybrid intelligent forecasting model based on empirical mode decomposition, support vector regression and adaptive linear neural network In Advances in natural computation, Springer: 2005; pp 324–327.

[14] Schmidt WF, Kraaijveld M, Duin RP. In *Feedforward neural networks with random weights*, Pattern Recognition, 1992. Vol. II. Conference B: Pattern Recognition Methodology and Systems, Proceedings., 11th IAPR International Conference on, 1992; IEEE: pp 1–4.

[15] Chen X, Dong ZY, Meng K, Xu Y, Wong KP, Ngan H. Electricity price forecasting with extreme learning machine and bootstrapping. Power Systems, IEEE Transactions on 2012, 27, 2055–2062.

[16] Nizar A, Dong Z, Wang Y. Power utility nontechnical loss analysis with extreme learning machine method. Power Systems, IEEE Transactions on 2008, 23, 946–955.

[17] Gao J, Wang Z, Yang Y, Zhang W, Tao C, Guan J, et al A novel approach for lie detection based on f-score and extreme learning machine. PloS one 2013, 8, e64704 doi: 10.1371/journal.pone.0064704 23755136 10.1371/journal.pone.0064704 PMC3670874

[18] Peng X, Lin P, Zhang T, Wang J. Extreme learning machine-based classification of adhd using brain structural mri data. PloS one 2013, 8, e79476 doi: 10.1371/journal.pone.0079476 24260229 10.1371/journal.pone.0079476 PMC3834213

[19] Blum A. Random projection, margins, kernels, and feature-selection In Subspace, latent structure and feature selection, Springer: 2006; pp 52–68.

[20] Balcan M-F, Blum A, Vempala S. Kernels as features: On kernels, margins, and low-dimensional mappings. Machine Learning 2006, 65, 79–94.

[21] Huang G-B, Zhou H, Ding X, Zhang R. Extreme learning machine for regression and multiclass classification. Systems, Man, and Cybernetics, Part B: Cybernetics, IEEE Transactions on 2012, 42, 513–529. 10.1109/TSMCB.2011.2168604 21984515

[22] Huang G-B. An insight into extreme learning machines: Random neurons, random features and kernels. Cognitive Computation 2014, 1–15.

[23] Pao Y-H, Takefji Y. Functional-link net computing. IEEE Computer Journal 1992, 25, 76–79.

[24] Huang G-B, Zhu Q-Y, Siew C-K. Extreme learning machine: Theory and applications. Neurocomputing 2006, 70, 489–501.

[25] Pao Y-H, Park G-H, Sobajic DJ. Learning and generalization characteristics of the random vector functional-link net. Neurocomputing 1994, 6, 163–180.

[26] Igelnik B, Pao Y-H. Stochastic choice of basis functions in adaptive function approximation and the functional-link net. Neural Networks, IEEE Transactions on 1995, 6, 1320–1329. 10.1109/72.471375 18263425

[27] Peng H, Long F, Ding C. Feature selection based on mutual information criteria of max-dependency, max-relevance, and min-redundancy. Pattern Analysis and Machine Intelligence, IEEE Transactions on 2005, 27, 1226–1238. 10.1109/TPAMI.2005.159 16119262

[28] Lian C, Zeng Z, Yao W, Tang H. Extreme learning machine for the displacement prediction of landslide under rainfall and reservoir level. Stochastic Environmental Research and Risk Assessment 2014, 28, 1957–1972.

[29] Frenzel S, Pompe B. Partial mutual information for coupling analysis of multivariate time series. Physical review letters 2007, 99, 204101 18233144 10.1103/PhysRevLett.99.204101

[30] Kraskov A, Stögbauer H, Grassberger P. Estimating mutual information. Physical review E 2004, 69, 066138. 10.1103/PhysRevE.69.066138 15244698

